# Complete genome sequence of *Kocuria rhizophila* BT304, isolated from the small intestine of castrated beef cattle

**DOI:** 10.1186/s13099-018-0270-9

**Published:** 2018-09-27

**Authors:** Tae Woong Whon, Hyun Sik Kim, Jin-Woo Bae

**Affiliations:** Department of Biology and Department of Life and Nanopharmaceutical Sciences, Kyung Hee University, 26 Kyungheedae-ro, Dongdaemun-gu, Seoul, 02447 Republic of Korea

**Keywords:** *Kocuria rhizophila* BT304, Complete genome sequence, Bovine small intestine, Branched chain amino acids

## Abstract

**Background:**

Members of the species *Kocuria rhizophila*, belonging to the family *Micrococcaceae* in the phylum *Actinobacteria*, have been isolated from a wide variety of natural sources, such as soil, freshwater, fish gut, and clinical specimens. *K*. *rhizophila* is important from an industrial viewpoint, because the bacterium grows rapidly with high cell density and exhibits robustness at various growth conditions. However, the bacterium is an opportunistic pathogen involved in human infections. Here, we sequenced and analyzed the genome of the *K*. *rhizophila* strain BT304, isolated from the small intestine of adult castrated beef cattle.

**Results:**

The genome of *K*. *rhizophila* BT304 consisted of a single circular chromosome of 2,763,150 bp with a GC content of 71.2%. The genome contained 2359 coding sequences, 51 tRNA genes, and 9 rRNA genes. Sequence annotations with the RAST server revealed many genes related to amino acid, carbohydrate, and protein metabolism. Moreover, the genome contained genes related to branched chain amino acid biosynthesis and degradation. Analysis of the OrthoANI values revealed that the genome has high similarity (> 97.8%) with other *K*. *rhizophila* strains, such as DC2201, FDAARGOS 302, and G2. Comparative genomic analysis further revealed that the antibiotic properties of *K*. *rhizophila* vary among the strains.

**Conclusion:**

The relatively small number of virulence-related genes and the great potential in production of host available nutrients suggest potential application of the BT304 strain as a probiotic in breeding beef cattle.

**Electronic supplementary material:**

The online version of this article (10.1186/s13099-018-0270-9) contains supplementary material, which is available to authorized users.

## Background

*Kocuria rhizophila*, a coccoid, Gram-positive, and spherical bacterium, was first identified by Kovacs et al. [1]. It belongs to the family *Micrococcaceae* in the phylum *Actinobacteria*, and has been isolated from a wide variety of natural sources such as soil [2], freshwater [3], and fish gut [4]. Considering its relatively small genome size among the order *Actinomycetales*, it is surprising that each *K*. *rhizophila* strain is highly adapted to its ecological niche. *K*. *rhizophila* is important from both ecological and industrial viewpoints, because the bacterium is tolerant to a wide range of organic solvents and capable of growing robustly in various conditions [2, 5]. More recently, attention has been drawn to clinical aspects of *K*. *rhizophila*. The clinical strains were isolated from blood samples of children with illness [6, 7], suggesting that the bacterium is an opportunistic pathogen involved in human infections. To date, nine strains of *K*. *rhizophila* are sequenced, with only two genomes completed. Here, we report the complete genome sequence of *K*. *rhizophila* BT304, isolated from the small intestine of castrated beef cattle. Genomic evaluation and comparative genomics may improve our understanding of the virulence factors and gut-associated symbiotic potential of *K*. *rhizophila*, and hence its suitability for probiotic application in the livestock industry.

## Methods

### Strain isolation and DNA extraction

*Kocuria rhizophila* BT304 was isolated from the small intestine of adult castrated beef cattle. The luminal content of the ileum was collected from a local slaughterhouse (Gunwi-Gun, South Korea). The isolate was cultivated on brain heart infusion (BHI) agar (Becton–Dickinson) in aerobic conditions at 37 °C for 24 h. Genomic DNA of the isolate was extracted using the MG Genomic DNA Purification Kit (MGmed) according to the manufacturer’s instruction. Total DNA was subjected to quality control by electrophoresis on a 1% agarose gel and quantified by a NanoDrop spectrophotometer (Thermo Scientific) and a Qubit fluorometer (Thermo Scientific).

### Whole genome sequencing, assembly, and gene annotation

The whole genome sequencing of *K*. *rhizophila* BT304 was performed using PacBio RS II (20-kb SMRTbell™ templates) and Illumina HiSeq4000 (TruSeq DNA PCR-Free 350-bp library) strategies. For obtaining 20-kb library, genomic DNA was sheared with g-TUBE (Covaris) and purified by AMPure PB magnetic beads (Beckman Coulter). The sequencing library for Illumina HiSeq4000 was prepared by random fragmentation of the DNA sample, followed by 5′ and 3′ adapter ligation. After filtering, we obtained 165,979 sub-reads with mean length of 8036-bp from the PacBio system. From the Illumina data set (34,363,358 raw reads), we obtained 12,131,322 filtered reads (quality score over 20 (Q20) = 99.12%). The reads were assembled using RS HGAP Assembly version 3.0 and polished with Quiver. The assembly of *K*. *rhizophila* BT304 was annotated using the RAST prokaryotic genome annotation server (http://rast.nmpdr.org/) [8], and the Position-Specific Iterative BLAST (PSI-BLAST) against the eggNOG version 4.5 database [9]. RNAmmer 1.2 [10] and tRNAscan-SE 1.21 [11] were used to identify rRNA and tRNA sequences, respectively. Prophage insert regions were detected with an on-line phage search tool, PHASTER [12].

### Phylogenetic analysis

We constructed a phylogenetic tree based on 16S rRNA gene sequences. Using the multiple sequence alignment program CLUSTAL W [13], the 16S rRNA gene sequences of the BT304 and related species were aligned. Phylogenetic trees were constructed using the following algorithms: neighbor-joining [14], maximum-parsimony [15] and maximum-likelihood [16] algorithms based on 1000 bootstrap replications in MEGA version 7 [17].

### Comparative genomic analysis

We performed comparative genomic analysis on nine *K*. *rhizophila* strains: DC2201, FDAARGOS 302, G2, D2, 14ASP, P7-4, TPW45, RF, and UMD0131. The reference genome sequences were downloaded from the NCBI genome database (http://www.ncbi.nlm.nih.gov/genome/). Average nucleotide identity (ANI) based on USEARCH (OrthoANIu) was used to assess overall similarity between two genome sequences [18]. Functional genes in each genome were predicted and annotated using the SEED subsystem in the RAST server. For whole genome comparison, the genome of *K*. *rhizophila* BT304 and other *K*. *rhizophila* genomes were aligned using the progressive MAUVE algorithm in the MAUVE multiple genome alignment software 2.4.0 [19].

### Quality assurance

A single colony of the *K*. *rhizophila* strain BT304 was repeatedly transferred to fresh BHI medium to obtain pure cultures. Before DNA extraction, the identity of the strain was verified through 16S rRNA gene sequencing. After the genome sequence was obtained, we confirmed the identity of the strain through a BLAST search of the 16S rRNA gene found in the genome.

## Results and discussion

### General features

**Fig. 1:**
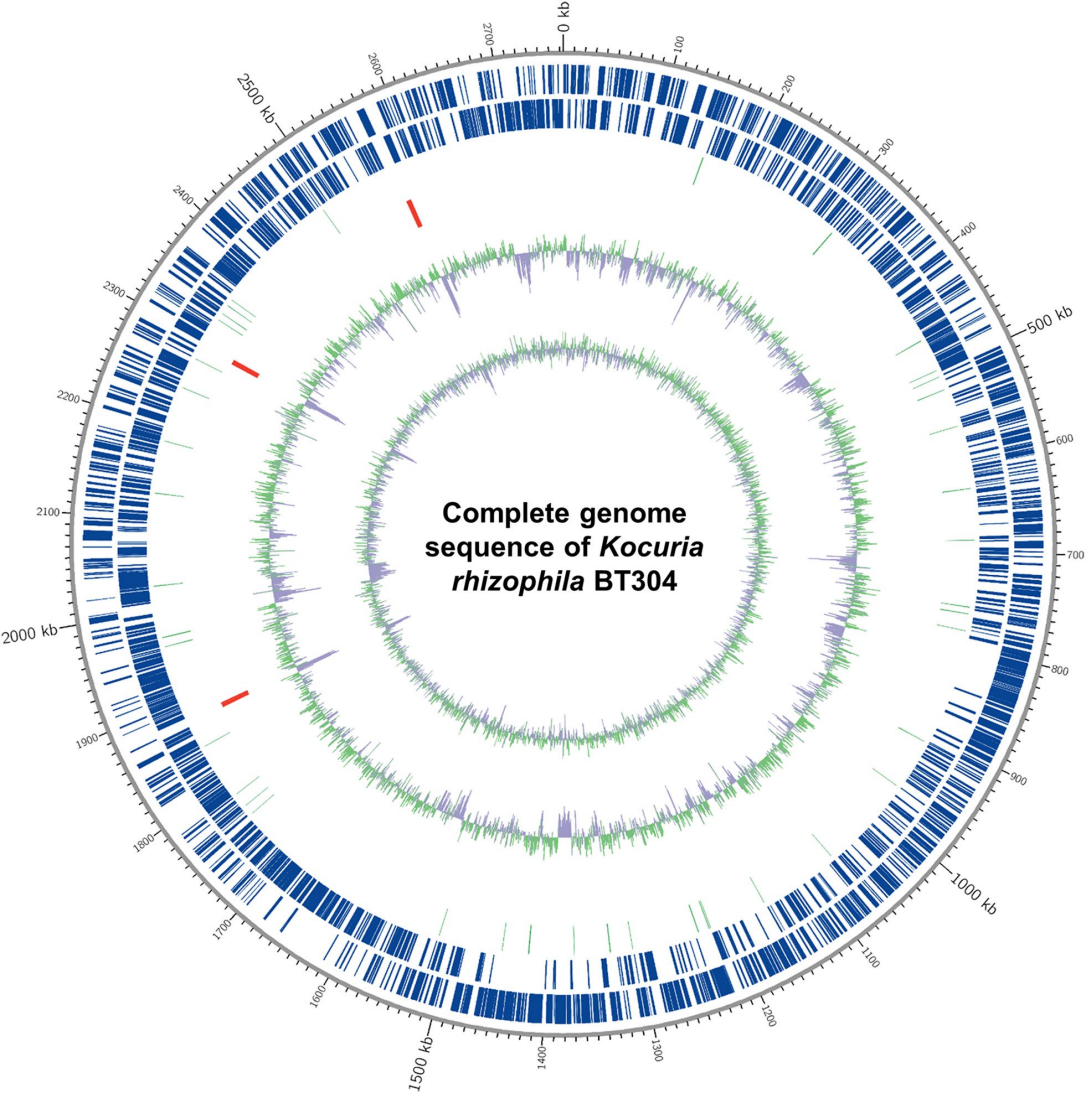
Graphic circular map of the *Kocuria rhizophila* BT304 genome. CDSs on the forward strand and CDSs on the reverse strand are indicated from the outer fringe to the center. Inner circles represent the tRNAs (light blue), rRNAs (red), GC content, and GC skew

**Fig. 2:**
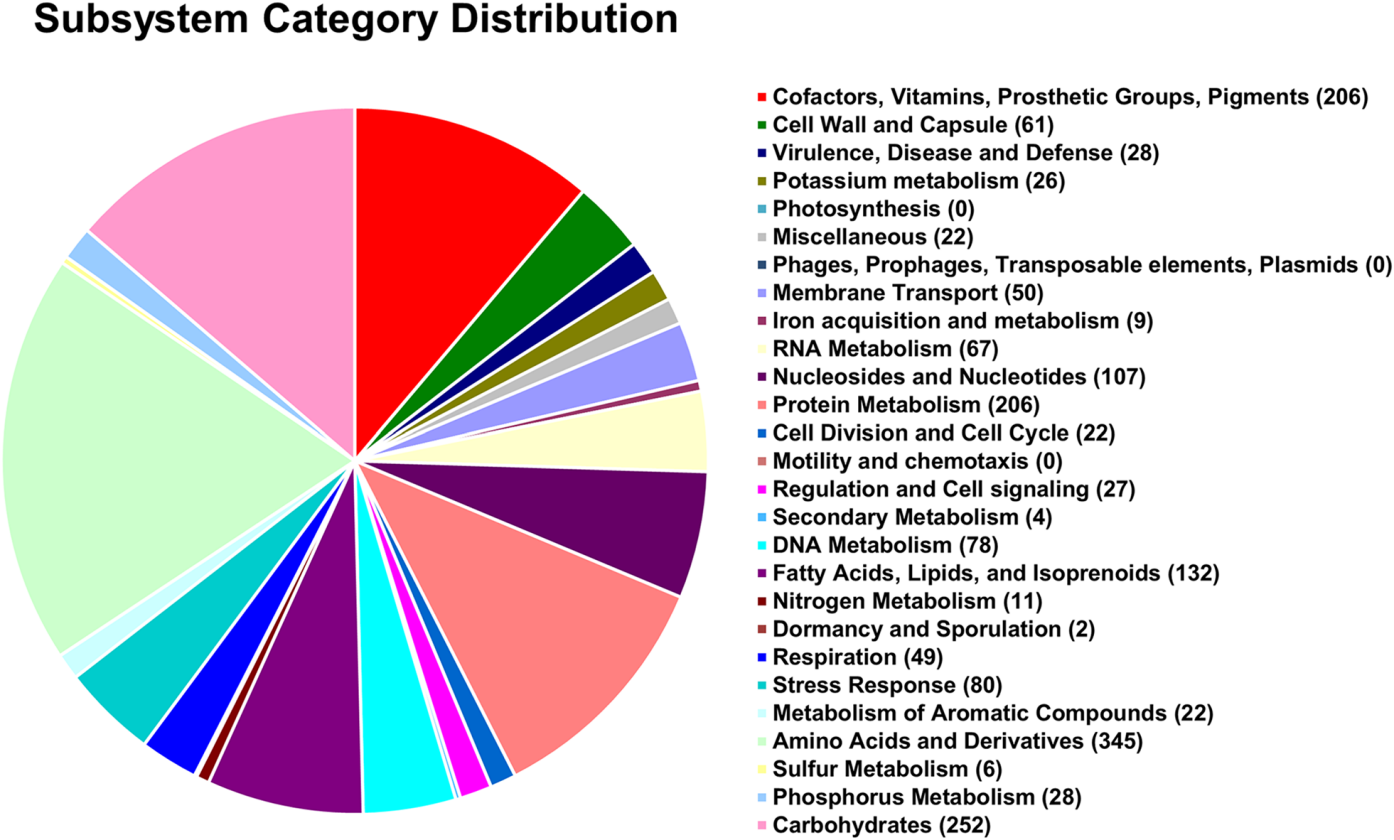
Analysis of annotated genes for the *Kocuria rhizophila* BT304 genome based on the SEED database. Of 2427 CDSs predicted by the RAST server, the subsystem coverage was 48%, contributing 361 subsystems

The genome coverage of the *K*. *rhizophila* strain BT304 was 329 fold, and the complete genome sequence consisted of a single circular chromosome of 2,763,150 bp. The genome contained 2359 coding sequences, 51 tRNA genes, and 9 rRNA genes. The circular map of the genome is displayed in Fig. 1, and detailed genomic features are listed in Table 1. GC content of the complete genome was 71.2%. The GC content and size of the BT304 chromosome was similar to those of other *K*. *rhizophila* strains, including *K*. *rhizophila* DC2201 (2,697,540 bp, 71.2% GC) [2], *K*. *rhizophila* P7-4 (2,820,331 bp, 70.5% GC) [4], and *K*. *rhizophila* TPW45 (2,701,701 bp, 70.6% GC) [3] (Table 2). The results of RAST annotation showed that the genome contained 2427 coding sequences with 55 RNA genes. In the SEED subsystem distribution, “amino acids and derivatives” (345 ORFs), “carbohydrates” (252 ORFs), “protein metabolism” (206 ORFs), and “cofactors, vitamins, prosthetic groups, pigments” (206 ORFs) were the most abundant categories (Fig. 2). Similar to the RAST annotation results, annotation of the BT304 genome using the eggNOG database revealed “amino acid transport and metabolism” (203 ORFs) as the most abundant category, followed by “replication, recombination and repair” (148 ORFs), and “translation, ribosomal structure and biogenesis” (145 ORFs) (Additional file 1: Table S1).

**Table 1:** Complete genome features of *Kocuria rhizophila* BT304

Item	Values
Sequencing platforms	PacBio RS II and Illumina HiSeq
Topology	Circular
Genome size (bp)	2,763,150
Genome coverage (fold)	329
DNA G + C (%)	71.2
tRNA genes	51
rRNA genes	9
Coding sequences	2359

**Table 2:** Comparison of the features of *Kocuria rhizophila* genomes

Attributes	*Kocuria rhizophila* genomes									
	BT304	DC2201	FDAARGOS302	G2	D2	14ASP	P7-4	TPW45	RF	UMB0131
Number of contigs	1	1	1	87	34	183	54	46	90	8
Size (bp)	2,763,150	2,697,540	2,697,877	2,881,857	2,636,961	2,698,103	2,820,331	2,701,701	2,778,506	2,833,440
GC (%)	71.2	71.2	71.2	70.8	70.8	70.8	70.5	70.6	70.6	70.5
OrthoANIu (%)	–	98.92	98.80	97.89	88.14	87.97	87.96	87.92	87.90	87.83
Resource	Bovine gut	Soil	N/A	Wall in slaughterhouse	Human feces	Soil	Fish gut	Freshwater	Soil	Human urine
Virulence, disease and defense	28	28	28	29	38	46	36	32	41	31
Resistance to fluoroquinolones	4	4	4	4	4	5	4	4	4	4
Resistance to vancomycin	1	1	1	1	–	–	–	–	1	–
Multidrug resistance efflux pumps	–	–	–	–	–	–	–	–	–	2
Amino acid and derivatives	345	356	356	350	322	351	318	316	323	318
BCAA biosynthesis	20	19	19	20	20	24	21	20	20	20
BCAA degradation	52	52	52	54	52	61	52	51	51	52

### Comparative genomic analysis

**Fig. 3:**
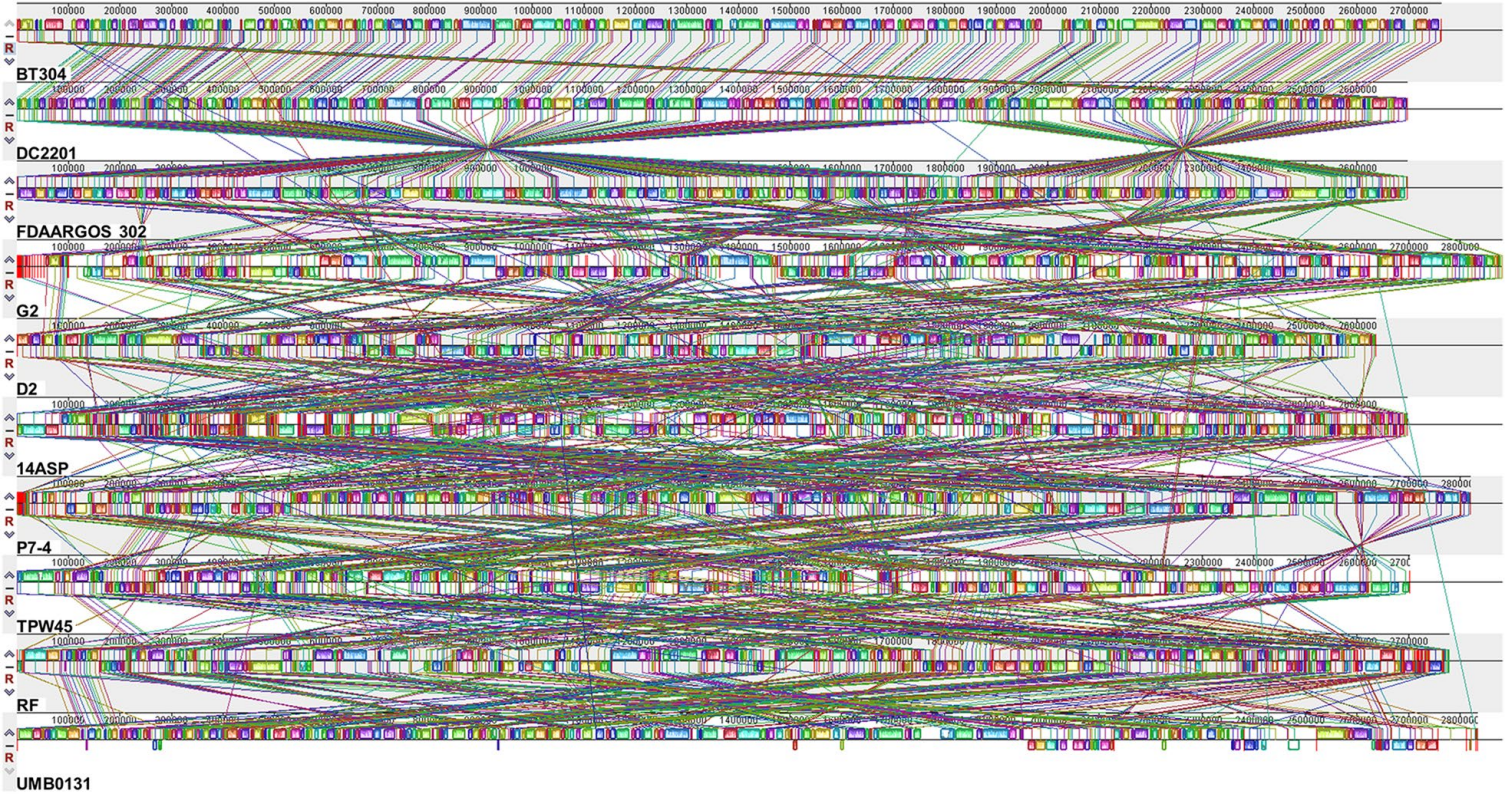
MAUVE alignment of the genome of *Kocuria rhizophila* BT304 and other *K*. *rhizophila* genomes. The locally collinear blocks shown with identical colors denote highly homologous regions. The genomes were drawn to scale based on the genome of *K*. *rhizophila* BT304

Based on 16S rRNA gene sequences, we constructed a phylogenetic tree to identify the phylogeny of the *K*. *rhizophila* strain BT304 within the family *Micrococcaceae* (Additional file 1: Figure S1). We compared the genome of *K*. *rhizophila* BT304 with those of nine other *K*. *rhizophila* strains. Analysis of the OrthoANI values revealed that the genome of strain BT304 is closest to that of *K*. *rhizophila* DC2201 (98.92% OrthoANI), followed by FDAARGOS 302 (98.80%), and G2 (97.89%) (Table 2). Between two genomes, an ANI value higher than 95–96% is regarded as the same species [20]. Accordingly, the strain BT304 was confirmed to be a species of *K*. *rhizophila*. Interestingly, however, the genome of BT304 showed much less genomic relatedness with several other *K*. *rhizophila* strains: D2 (88.14%), 14ASP (87.97%), P7-4 (87.96%), TPW45 (87.92%), RF (87.90%), and UMD0131 (87.83%). We next conducted whole genome comparison of the genome of *K*. *rhizophila* BT304 with other publically available *K*. *rhizophila* genomes based on the MAUVE multiple genome alignment. The locally collinear blocks between the BT304 and DC2201 genomes were highly homologous, whereas considerable differences in genomic regions were observed between the BT304 and other *K*. *rhizophila* genomes (Fig. 3). These results imply that each *K*. *rhizophila* strain can adapt to its respective ecological niche by gaining and/or losing genomic elements.

### Virulence-related factors

Because several strains belonging to the species *K*. *rhizophila* have been isolated from clinical specimens (i.e., human blood) [6, 7], we evaluated virulence-related factors from the genome of strain BT304 and other publically available *K*. *rhizophila* genomes. Based on comparison with the SEED database, we found 28–46 CDSs annotated as the “virulence, disease and defense” category from the *K*. *rhizophila* genomes (Table 2). The BT304 strain possessed the lowest number of virulence-related genes (28 CDSs), whereas the 14ASP strain (isolated from soil) contained the highest number of virulence-related genes (46 CDSs). To determine whether a degree of virulence-related factors observed from the strain BT304 is suitable for probiotic applications, we accessed the number of virulence-related genes in genomes of eight commercially available probiotic strains [21]. The probiotic strains mainly composed of the genera *Bifidobacterium* and *Lactobacillus* contained 22–47 CDSs assigned to the “virulence, disease and defense” category (Additional file 1: Table S2).

We next assessed antibiotic resistant potential in the *K*. *rhizophila* genomes. Genes related to the sub-category “resistance to fluoroquinolones” were found in the genomes of all *K*. *rhizophila* strains. Several strains (e.g., BT304, DC2201, FDAARGOS 302, G2, and RF) contained genes related to “resistance to vancomycin”. Only the UMB0131 strain isolated from human urine possessed genes related to “multidrug resistance efflux pumps”. Collectively, our comparative analysis suggested that the antibiotic properties of *K*. *rhizophila* vary among the strains.

### Prophage insertion

To identify prophage contamination in the genome of *K*. *rhizophila* BT304, we conducted PHAST phage search analysis. The genome contained four incomplete prophages at positions 876,510–884,942, 1,118,199–1,125,394, 1,367,325–1,389,936, and 2,021,003–2,029,604 bp (Additional file 1: Table S3). PHAST analysis in other *K*. *rhizophila* strains further revealed that the number of prophages varies among the strains. Strains with an ANI of over 97% with the strain BT304 were predicted to have 1–3 incomplete prophages, whereas the remaining strains contained no prophage. The strain P7-4, isolated from the fish gut, was the only exception, containing one incomplete prophage.

### Branched chain amino acid metabolism

The BT304 strain was isolated from the bovine small intestine where dietary nutrients (e.g., amino acids, lipids, and carbohydrates) are metabolized by abundant microbiota [22, 23]. As described above, the BT304 strain possessed a relatively small genome, but many genes related to amino acid metabolism (Fig. 2 and Additional file 1: Table S1). Based on comparison with the SEED database, we found a relatively similar number of genes (318–356 CDSs) annotated as the “amino acid and derivatives” category across the *K*. *rhizophila* genomes (Table 2), suggesting that *K*. *rhizophila* can utilize dietary amino acids, and might be an indigenous member of the small intestinal gut microbiota in beef cattle.

In mammals, including human and mouse, branched chain amino acids (BCAAs) are responsible for lipogenesis in adipocytes [24] and lipid accumulation in skeletal muscles [25]. Therefore, accumulation of BCAAs is regarded as a key risk factor leading to obesity. However, these metabolic aspects of BCAAs would be beneficial in economical feeding of breeding animals. Annotating the genome of BT304 using the SEED database revealed genes related to both BCAA biosynthesis (20 CDSs) and degradation (52 CDSs) (Table 2). Comparative genomic analysis showed that other reference *K*. *rhizophila* genomes also contain more than 70 genes related to BCAA metabolism.

## Conclusions

We described the complete genome sequence of *K*. *rhizophila* BT304, isolated from the small intestine of castrated beef cattle. Our genomic analysis suggests that the relatively small number of virulence-related genes in combination with the potential for the production of host available nutrients make the *K*. *rhizophila* strain BT304 a probiotic candidate in breeding beef cattle.

## Additional file

**Additional file 1: Figure S1.** Phylogenetic tree based on 16S rRNA gene sequences, reconstructed with the neighbour-joining (NJ), maximum-parsimony (MP) and maximum-likelihood (ML) algorithms, indicating the taxonomic positions of strain BT304 and close relatives in the family *Micrococcaceae*. **Table S1.** Analysis of annotated genes for the *Kocuria rhizophila* BT304 genome based on the eggNOG database. **Table S2.** Comparison of the virulence related factors in commercially available probiotics. **Table S3.** Phage sequences found in *Kocuria rhizophila* genomes.

## Abbreviations

ANI: average nucleotide identity
CDS: coding DNA sequence
ORF: open reading frame
BCAA: branched chain amino acid
