# Supplementary material for: Complete genome sequence of Kocuria rhizophila BT304, isolated from the small intestine of castrated beef cattle
Source: Gut Pathog. 2018 Sep 27;10:42. doi: 10.1186/s13099-018-0270-9 (PMC6161389; doi:10.1186/s13099-018-0270-9)
Supplement: Supplementary file 1 — Additional file 1: Figure S1. Phylogenetic tree based on 16S rRNA gene sequences, reconstructed with the neighbour-joining (NJ), maximum-parsimony (MP) and maximum-likelihood (ML) algorithms, indicating the taxonomic positions of strain BT304 and close relatives in the family Micrococcaceae. Table S1. Analysis of annotated genes for the Kocuria rhizophila BT304 genome based on the eggNOG database. Table S2. Comparison of the virulence related factors in commercially available probiotics. Table S3. Phage sequences found in Kocuria rhizophila genomes. [file 13099_2018_270_MOESM1_ESM.docx]

**Additional file 1**

**Complete genome sequence of *Kocuria rhizophila* BT304, isolated from the small intestine of castrated beef cattle**

Tae Woong Whon^†^, Hyun Sik Kim^†^, and Jin-Woo Bae*

Department of Biology and Department of Life and Nanopharmaceutical Sciences, Kyung Hee University, 26 Kyungheedae-ro, Dongdaemun-gu, Seoul 02447, Republic of Korea

^†^Equal contributors

*Correspondence: **baejw@khu.ac.kr**; Tel: +82-2-961-2312


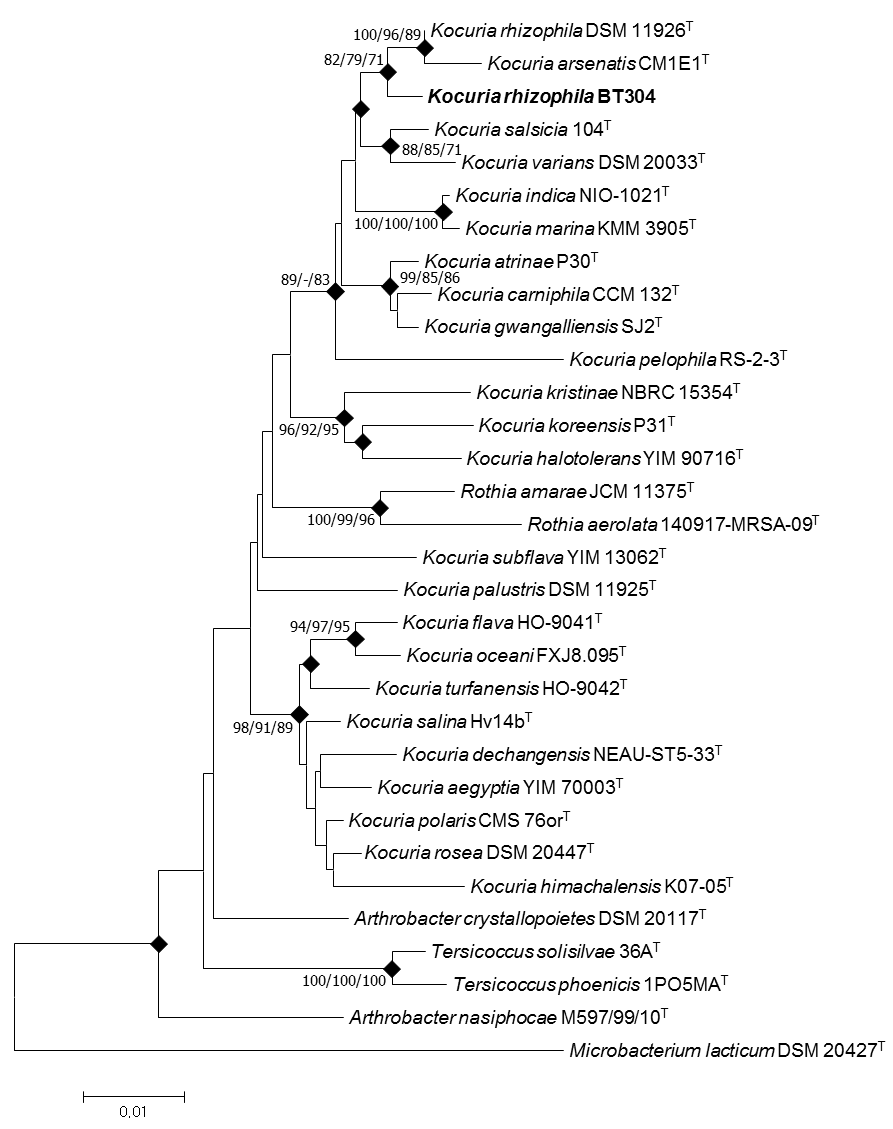


**Fig. S1.** Phylogenetic tree based on 16S rRNA gene sequences, reconstructed with the neighbour-joining (NJ), maximum-parsimony (MP) and maximum-likelihood (ML) algorithms, indicating the taxonomic positions of strain BT304 and close relatives in the family *Micrococcaceae*. Diamonds indicate communal branches in the phylogenetic trees generated using the three algorithms (NJ, MP and ML). Bootstrap values shown at nodes are expressed as a percentage of 1000 replicates. Values <70 % are not shown. *Microbacterium lacticum* DSM 20427^T^ was used as an out-group. Bar indicates 0.01 substitutions per nucleotide.

**Table S1.** Analysis of annotated genes for the *Kocuria rhizophila* BT304 genome based on the eggNOG database.

| **eggNOG** | **Description** | **Count** |
| --- | --- | --- |
| J | Translation, ribosomal structure and biogenesis | 145 |
| A | RNA processing and modification | 1 |
| K | Transcription | 133 |
| L | Replication, recombination and repair | 148 |
| B | Chromatin structure and dynamics | 1 |
| D | Cell cycle control, cell division, chromosome partitioning | 16 |
| Y | Nuclear structure | 0 |
| V | Defense mechanisms | 27 |
| T | Signal transduction mechanisms | 58 |
| M | Cell wall/membrane/envelope biogenesis | 95 |
| N | Cell motility | 0 |
| Z | Cytoskeleton | 0 |
| W | Extracellular structures | 0 |
| U | Intracellular trafficking, secretion, and vesicular transport | 23 |
| O | Posttranslational modification, protein turnover, chaperones | 68 |
| C | Energy production and conversion | 130 |
| G | Carbohydrate transport and metabolism | 130 |
| E | Amino acid transport and metabolism | 203 |
| F | Nucleotide transport and metabolism | 77 |
| H | Coenzyme transport and metabolism | 81 |
| I | Lipid transport and metabolism | 85 |
| P | Inorganic ion transport and metabolism | 122 |
| Q | Secondary metabolites biosynthesis, transport and catabolism | 30 |
| R | General function prediction only | 165 |
| S | Function unknown | 615 |
| Total | - | 2,353 |

**Table S2.** Comparison of the virulence related factors in commercially available probiotics.

| **Genome** | **Virulence, disease and defense (count)** |
| --- | --- |
| BT304 | 28 |
| *Bifidobacterium animalis* subsp. *lactis* BL03 | 22 |
| *Bifidobacterium animalis* subsp. *lactis* BI04 | 22 |
| *Bifidobacterium breve* BB02 | 37 |
| *Lactobacillus acidophilus* BA05 | 35 |
| *Lactobacillus helveticus* BD08 | 47 |
| *Lactobacillus paracasei* BP07 | 31 |
| *Lactobacillus plantarum* BP06 | 39 |
| *Streptococcus thermophilus* BT01 | 25 |

**Table S3.** Phage sequences found in *Kocuria rhizophila* genomes.

| **Genome** | **Region length** | **Completeness** | **Score** | **Total proteins** | **Region position** | **Most common phage** | **GC %** |
| --- | --- | --- | --- | --- | --- | --- | --- |
| BT304 | 8.4 Kb | Incomplete | 10 | 6 | 876,510–884,942 | PHAGE_Bacill_G_NC_023719(2) | 70.82 |
|  | 7.1 Kb | Incomplete | 10 | 7 | 1,118,199–1,125,394 | PHAGE_Bacill_G_NC_023719(2) | 71.00 |
|  | 22.6 Kb | Incomplete | 40 | 10 | 1,367,325–1,389,936 | PHAGE_Gordon_Terrapin_NC_031001(3) | 65.01 |
|  | 8.6 Kb | Incomplete | 10 | 9 | 2,021,003–2,029,604 | PHAGE_Flavob_1H_NC_031911(6) | 68.44 |
| DC2201 | 8.4 Kb | Incomplete | 10 | 6 | 730,903–739,336 | PHAGE_Bacill_G_NC_023719(2) | 70.76 |
|  | 6.2 Kb | Incomplete | 10 | 7 | 1,012,249–1,018,465 | PHAGE_Bacill_G_NC_023719(2) | 70.24 |
|  | 8.5 Kb | Incomplete | 10 | 11 | 2,540,467–2,548,975 | PHAGE_Pseudo_OBP_NC_016571(1) | 71.43 |
| FDAARGOS  302 | 6.2 Kb | Incomplete | 10 | 7 | 808,998–815,214 | PHAGE_Bacill_G_NC_023719(2) | 70.24 |
|  | 8.4 Kb | Incomplete | 10 | 6 | 1,088,116–1,096,549 | PHAGE_Bacill_G_NC_023719(2) | 70.76 |
|  | 8.5 Kb | Incomplete | 20 | 10 | 1,976,104–1,984,612 | PHAGE_Cronob_vB_CsaM_GAP32_NC_019401(1) | 71.43 |
| G2 | 7 Kb | Incomplete | 40 | 12 | 65,220–72,290 | PHAGE_Salmon_SJ46_NC_031129(1) | 63.89 |
| D2 | - | - | - | - | - | - | - |
| 14ASP | - | - | - | - | - | - | - |
| P7-4 | 9.1 Kb | Incomplete | 10 | 7 | 212,171–221,338 | PHAGE_Mycoba_Xeno_NC_031243(4) | 69.07 |
| TPW45 | - | - | - | - | - | - | - |
| RF | - | - | - | - | - | - | - |
| UMB0131 | - | - | - | - | - | - | - |
